# Quantitative comparison of PET performance—Siemens Biograph mCT and mMR

**DOI:** 10.1186/s40658-016-0142-7

**Published:** 2016-02-25

**Authors:** Anna M. Karlberg, Oddbjørn Sæther, Live Eikenes, Pål Erik Goa

**Affiliations:** Department of Radiology and Nuclear Medicine, St. Olavs Hospital, Olav Kyrres gt 17, 7006 Trondheim, Norway; Department of Circulation and Medical Imaging, Norwegian University of Science and Technology, Postbox 8905, 7491 Trondheim, Norway; Department of Physics, Norwegian University of Science and Technology, Trondheim, Norway

**Keywords:** PET performance, Quantitative image quality, PET/CT, PET/MR

## Abstract

**Background:**

Integrated clinical whole-body PET/MR systems were introduced in 2010. In order to bring this technology into clinical usage, it is of great importance to compare the performance with the well-established PET/CT. The aim of this study was to evaluate PET performance, with focus on image quality, on Siemens Biograph mMR (PET/MR) and Siemens Biograph mCT (PET/CT).

**Methods:**

A direct quantitative comparison of the performance characteristics between the mMR and mCT system was performed according to National Electrical Manufacturers Association (NEMA) NU 2-2007 protocol. Spatial resolution, sensitivity, count rate and image quality were evaluated. The evaluation was supplemented with additional standardized uptake value (SUV) measurements.

**Results:**

The spatial resolution was similar for the two systems. Average sensitivity was higher for the mMR (13.3 kcps/MBq) compared to the mCT system (10.0 kcps/MBq). Peak noise equivalent count rate (NECR) was slightly higher for the mMR (196 kcps @ 24.4 kBq/mL) compared to the mCT (186 kcps @ 30.1 kBq/mL). Scatter fractions in the clinical activity concentration range yielded lower values for the mCT (34.9 %) compared to those for the mMR (37.0 %). Best image quality of the systems resulted in approximately the same mean hot sphere contrast and a difference of 19 percentage points (pp) in mean cold contrast, in favour of the mCT. In general, point spread function (PSF) increased hot contrast and time of flight (TOF) increased both hot and cold contrast. Highest hot contrast for the smallest sphere (10 mm) was achieved with the combination of TOF and PSF on the mCT. Lung residual error was higher for the mMR (22 %) than that for the mCT (17 %), with no effect of PSF. With TOF, lung residual error was reduced to 8 % (mCT). SUV was accurate for both systems, but PSF caused overestimations for the 13-, 17- and 22-mm spheres.

**Conclusions:**

Both systems proved good performance characteristics, and the PET image quality of the mMR was close to that of the mCT. Differences between the systems were mainly due to the TOF possibility on the mCT, which resulted in an overall better image quality, especially for the most challenging settings with higher background activity and small uptake volumes.

## Background

Whole-body hybrid PET/MR technology is a promising and powerful tool with several potential advantages compared to the more established PET/CT. PET/MR has the ability to perform simultaneous PET and MR data acquisitions, and MR offers functional-imaging capabilities as well as better contrast among soft tissues than CT imaging. Also, the significant radiation dose to the patient, contributed from a CT scan, can be excluded [1]. There are a number of differences in the PET system design as well as in the acquisition and reconstruction methods between this later introduced modality and the PET/CT system. The main technological differences are the design of the PET detectors and the attenuation correction (AC) of PET data. Traditional photomultiplier tubes (PMTs) are incompatible with the strong magnetic field in a PET/MR system. Several approaches have been applied to solve this problem. In Siemens Biograph mMR, avalanche photodiodes (APDs) are used in PET detectors instead of PMTs. Due to the relatively low timing resolution of APDs, the Siemens mMR lacks time-of-flight (TOF) capability. TOF option is however available in the Philips Ingenuity TF PET/MR non-integrated system as well as in GEs integrated Signa PET/MR with silicon-PM detector technology. TOF is known to improve image quality due to increased signal-to-noise ratio [2].

For accurate quantification of the radionuclide uptake, PET data need to be corrected for photon attenuation. Attenuation information can be obtained from either a transmission scan in stand-alone PET or derived from CT images in combined PET/CT systems [3]. Low-dose CT scans are today’s standard for AC in PET/CT imaging. Alternative methods have been proposed for AC in PET/MR, since MR images do not reflect electron densities. These methods can be classified into three main categories: emission, segmentation and atlas-based approaches [4]. Siemens Biograph mMR uses a segmentation-based technique derived from an MR sequence as basis for AC in clinical procedures. However, conventional MR sequences do not image bone tissue, and this becomes a source of error in clinical PET AC images from a PET/MR system. For phantom studies, vendor-provided attenuation maps (CT-based templates) can be used instead of MR-based attenuation maps. Artefacts that usually occur when scanning large water-filled volumes are then avoided, and attenuation in phantom housing is accounted for.

It is essential to evaluate the performance of PET/MR in comparison to PET/CT, with respect to image quality and quantitative parameters. Several studies have compared the PET images from PET/CT and PET/MR in clinical data [5–17], and the vast majority have demonstrated equal diagnostic performance and detection rates, despite some differences in quantification. However, attenuation correction issues in clinical data will always lead to quantification inaccuracies in such comparisons. Biological factors will also influence the activity distribution within a patient between the PET/CT and PET/MR examination. By using a phantom and CT-based attenuation correction for both PET/CT and PET/MR, such limiting factors are eliminated.

Previously, full scanner performance measurements, according to National Electrical Manufacturers Association (NEMA) NU 2-2007 protocol, have been performed by Delso et al. [18] for the Siemens Biograph mMR system and by Jakoby et al. [19] and Marti-Climent et al. [20] for the Siemens Biograph mCT. To the best of our knowledge, no direct phantom comparison between the two systems has been reported, and this is important to establish a basis on which a further comparison and discussion of clinical performance can be made.

The main purpose of this study was to perform a direct quantitative comparison of PET performance, with main focus on PET image quality, between state-of-the art PET/CT and the later introduced PET/MR, using the same physical phantoms, same phantom preparation for image quality, identical data analysis and the same measurement protocol (NEMA NU 2-2007).

## Methods

### Systems

The Siemens Biograph mCT (software version syngo MI.PET/CT 2012A) (Siemens Healthcare, Erlangen, Germany) consists of a PET detector with 4 rings, 48 detector blocks in each ring and lutetium oxyorthosilicate (LSO) crystals of 4 × 4 × 20 mm in a 13 × 13 array coupled to a 2 × 2 PMT array in each detector block. This gives an axial PET field of view (FOV) of 22.1 cm. The transaxial FOV is 70 cm. Detector ring diameter is 84.2 cm. Time coincidence window is 4.1 ns, system time resolution 540 ps and energy window 435–650 keV. An integrated 64-slice CT is used for attenuation correction of PET data [21].

The Siemens Biograph mMR (software version B20P) (Siemens Healthcare, Erlangen, Germany) consists of a PET detector with 8 rings, 56 detector blocks in each ring and LSO crystals of 4 × 4 × 20 mm in a 8 × 8 array coupled to a 3 × 3 APD array in each detector block. This gives an axial PET FOV of 25.8 cm. The transaxial FOV is 58.8 cm. Time coincidence window is 5.86 ns and the energy window 430–610 keV. The integrated whole-body MR is a 3 Tesla (T) niobium-titanium magnet with 60-cm patient bore diameter [22].

### Performance measurements

Performance measurements included spatial resolution, sensitivity, count rate performance, scatter fraction and image quality for both systems. All measurements were performed according to the NEMA NU 2-2007 protocol and evaluated with Siemens NEMA 2007 software for both systems. Cross calibration (between dose calibrator and systems and between the systems) and normalization with time alignment and full detector calibration have been performed on a regular basis according to manufacturer’s recommendations on both scanners. To assure good quality, this was also done prior to this study.

### Spatial resolution

The spatial resolution of the systems was measured by using a capillary tube with ^18^F^−^-absorbing resin as a point source (start activities, 7.0 MBq (mCT), 8.9 MBq (mMR); activity concentrations at scan start, 3.8 GBq/mL (mCT), 2.9 GBq/mL (mMR)) placed at six different positions within the PET FOV ((*X*,*Y*,*Z*) = (0,1,1/2FOV_z_), (0,1,3/4FOV_z_), (10,0,3/4FOV_z_), (10,0,1/2FOV_z_), (0,10,1/2FOV_z_) and (0,10,3/4FOV_z_) cm). The point source was placed on an L-fixture and accurately aligned with the gantry lasers and with the semiautomatic positioning software included in the Siemens NEMA software. Average net trues were 2.8 million counts for the mCT and 2.3 million counts for the mMR. Sinogram data was reconstructed using Fourier rebinning (FORE) algorithm and filtered back projection (FBP) into image matrices of 400 × 400 × 109 voxels/2.04 × 2.04 × 2.03 mm (mCT) and 344 × 344 × 127 voxels/2.09 × 2.09 × 2.03 mm (mMR). It can be noted that Siemens NEMA test instructions accept a larger point source volume than the NEMA NU 2-2007 instructions. Therefore, a minor compromise in volume was used to be able to perform the tests.

### Sensitivity

Sensitivity was measured using a 70-cm line source of known ^18^F^−^-activity inserted into the five standardized NEMA aluminium sleeves at two positions in the PET FOV (*X*,*Y* = (0,0) and (0,10) cm). Start activities were 8.4 MBq (0 cm) and 6.7 MBq (10 cm) for the mCT and 12.3 MBq (0 cm) and 9.8 MBq (10 cm) for the mMR. Acquisitions included five measurements for each point, 300 s each. For each measurement, one sleeve was removed. By changing the amount of attenuation surrounding the line source, the sensitivity can be extrapolated to zero attenuated medium for each position and the average sensitivity for each system can be calculated. The data was decay corrected.

### Count rate

Count rate measurements were performed utilizing a high activity line source (^18^F^−^) in the NEMA scatter phantom, centered in the transverse and axial PET FOV. Start activity was 997 MBq (mCT) and 627 MBq (mMR), which is high enough to allow peak NECR estimation for the systems. Regular 600-s measurements were taken until activity approached zero (14.7 h for both systems). Sinogram data was used to estimate NECR and scatter fractions of the systems for varying activity concentrations within the FOV. NECRs were calculated using delayed coincidence window for randoms subtraction.

Count rate accuracy was estimated by extrapolating the true rate for low-activity concentrations (where count losses and randoms can be effectively neglected) back to higher activity levels and comparing it to the measured and corrected trues rate. Data was reconstructed using the FORE algorithm and FBP.

### Image quality

**Fig. 1:**
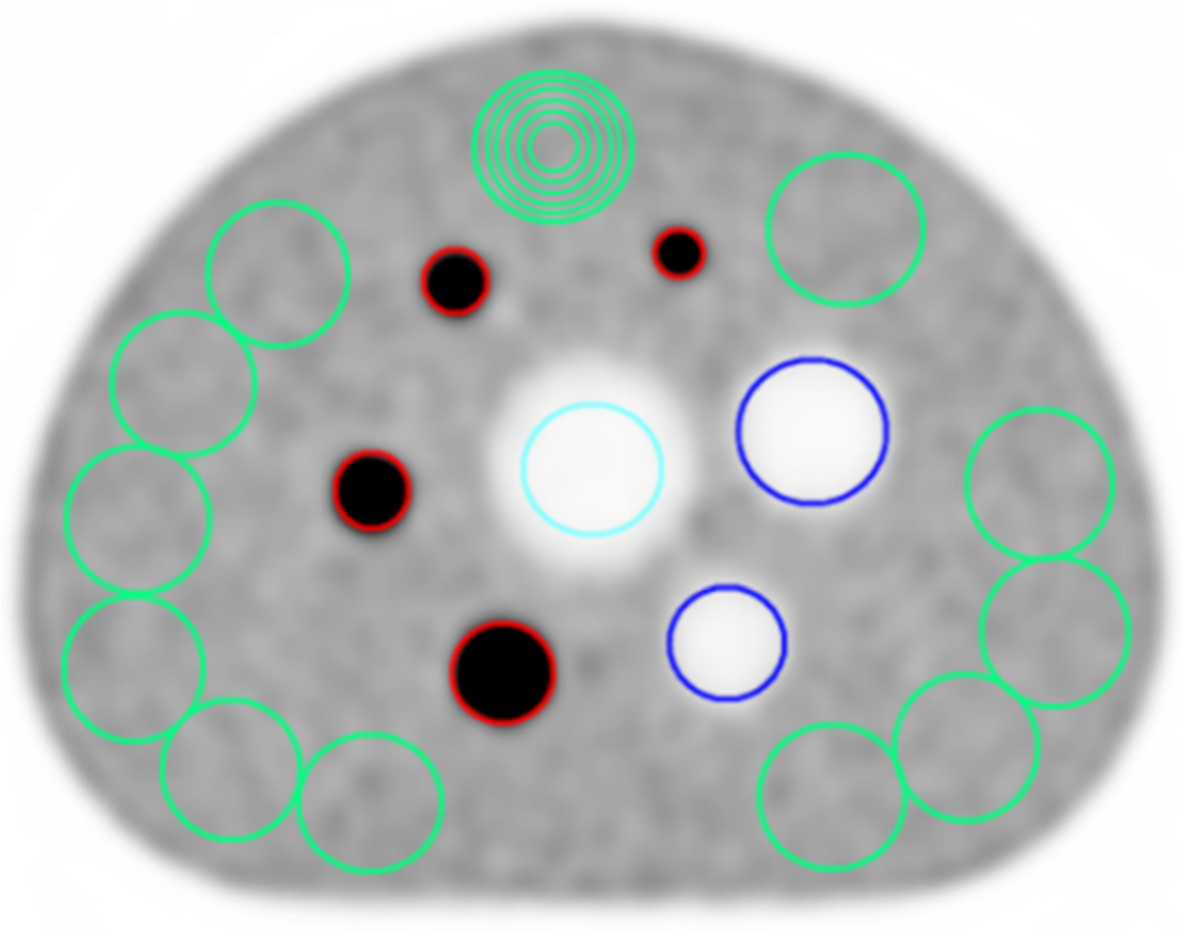
NEMA phantom ROIs. Typical ROIs drawn by Siemens NEMA image quality software over hot (*red*) and cold (*dark blue*) spheres, lung insert (*light blue*) and background (*green*)

The PET NEMA image quality phantom (PTW, Freiburg, Germany) was prepared with ^18^F^−^ once for each background-to-hot sphere activity concentration (1:8 and 1:4) as a part of the NEMA NU 2-2007 procedure [23], which was the NEMA protocol employed by the manufacturer at the time of these experiments. Background activity at scan start, mCT, was 5.39 kBq/mL (1:8) and 5.04 kBq/mL (1:4), respectively. The phantom contained hot spheres (inner diameters 10, 13, 17 and 22 mm), cold spheres (28 and 37 mm inner diameter) and a lung insert (50 mm diameter). The NEMA scatter phantom, with a line source of 115 MBq (at scan start mCT), was placed next to the image quality phantom in the axial direction to simulate activity from a patient’s body parts outside FOV. The acquisitions were performed on the mCT followed by the mMR (time delay 1 h) scanner for both activity concentrations, leaving background activity concentrations of 3.69 kBq/mL (1:8) and 3.47 kBq/mL (1:4) at scan start mMR. Acquisition times were 9 min (mCT) and 12 min (mMR) according to manufacturer’s specifications. This resulted in 145 Mcts for the mCT and 186 Mcts for the mMR in total number net trues for the 1:8 concentration, a difference of 28 % in favour of the mMR, with the same relation for the 1:4 concentration. Iterative reconstruction (3D ordered subset expectation, maximization (OSEM) algorithm) was employed, with/without point spread function (PSF) correction and for the mCT additionally with TOF. A matrix size of 400 × 400 and 4-mm Gaussian filter were used on the mCT. Corresponding settings on the mMR were 344 × 344 matrix and 4-mm Gaussian filter. This corresponds to an isotropic voxel size of ~2 mm in the reconstructed images from both systems to make the best possible comparison. In all comparisons, the same number of iterations (2–8) and subsets (21) were used (except from the mCT 3D OSEM reconstructions, where 24 subsets were used). For direct comparison of image contrast and standardized uptake values (SUV), reconstructions were done with three iterations. This number of iterations was chosen based on image quality analysis for the smallest sphere and the most challenging phantom preparation (1:4 activity concentration). Image quality parameters, such as hot and cold sphere contrast, lung residual error and background variability were calculated according to NEMA NU 2-2007 standard using Siemens NEMA image quality software for both modalities. This software automatically delineated regions of interest (ROIs) in five image planes (Fig. 1). A total of 60 background ROIs of each sphere size were applied (12 per 5 planes), i.e. ROIs of the same sizes as the smaller spheres (10, 13, 17, 22 and 28 mm) were applied concentric to each of the 37-mm ROIs on the background [23].

European Association of Nuclear Medicine (EANM) procedure guidelines [24] recommend reporting SUV_max_ and at least one additional SUV based on threshold, or peak value, for 2D or 3D clinical data. Therefore, SUV_max_, SUV_50_ and SUV_80_ were evaluated for all hot spheres and for both background concentrations, using Siemens Syngo.via workstation for the 3D volumes.

### Corrections in image quality measurements

AC in mCT data was based on a low-dose CT scan (120 kV; reference mAs, 40; pitch, 0.95; rotation time, 0.5 s; collimation, 64 × 0.6 mm; slice width, 3 mm.

Water-filled phantoms may induce strong artefacts in MR imaging at magnetic field strengths above 1.5 T, and MR-based AC is therefore not recommended for large phantoms at 3 T. The artefacts are caused by inhomogeneous radiofrequency excitation when fluids with high relative permittivity are imaged [25]. In addition, phantom housing are not included in MR-based AC. Ziegler et al. [26] and Boellard et al [27] have demonstrated that CT-based AC is superior to MR-based AC for image quality evaluation of the NEMA phantom in integrated PET/MR. In accordance with that, AC for the NEMA phantom in this study was performed with a vendor-provided attenuation map (μ-map) on the mMR system (CT-based template). Linear attenuation coefficients were 0.095 cm^−1^ for water and 0.026 cm^−1^ for the lung insert (same as the mean attenuation values derived from the low-dose CT scan used for AC of mCT data).

All reconstructions included corrections for attenuation, detector normalization, randoms and scatter. Truncation compensation was automatically not used for the PET/CT scans and was disabled for the PET/MR scans, since the phantom did not exceed the FOV.

## Results

### Spatial resolution, sensitivity and count rate performance

The performance characteristics of the systems, including spatial resolution, sensitivity, NECR, scatter fraction and count rate accuracy, are summarized in Table 1. With the applied reconstruction method, the spatial resolution characteristics were similar for the two systems. The average sensitivity was higher for the mMR system compared to the mCT system. Peak NECR was slightly higher for the mMR system and was reached at a lower activity concentration compared to the mCT. Scatter fractions could be considered similar at peak NECR.

**Table 1:** Performance characteristics of the Biograph mMR and Biograph mCT

Parameter	mMR	mCT
Resolution (mm)		
Axial FWHM/FWTM @ 1 cm	4.1/8.2	4.4/8.8
Transverse FWHM/FWTM @ 1 cm	4.0/8.0	4.4/8.3
Axial FWHM/FWTM @ 10 cm	6.4/11.8	5.7/10.7
Transverse FWHM/FWTM @ 10 cm	5.0/10.8	4.9/9.3
Average sensitivity (kcps/MBq)	13.3	10.0
Peak NECR (kcps)	196 @ 24.4 kBq/mL	186 @ 30.1 kBq/mL
Scatter fraction (peak NECR)	37.9 %	37.7 %
Count rate accuracy (mean bias, peak NECR)	4.9 %	1.9 %

**Fig. 2:**
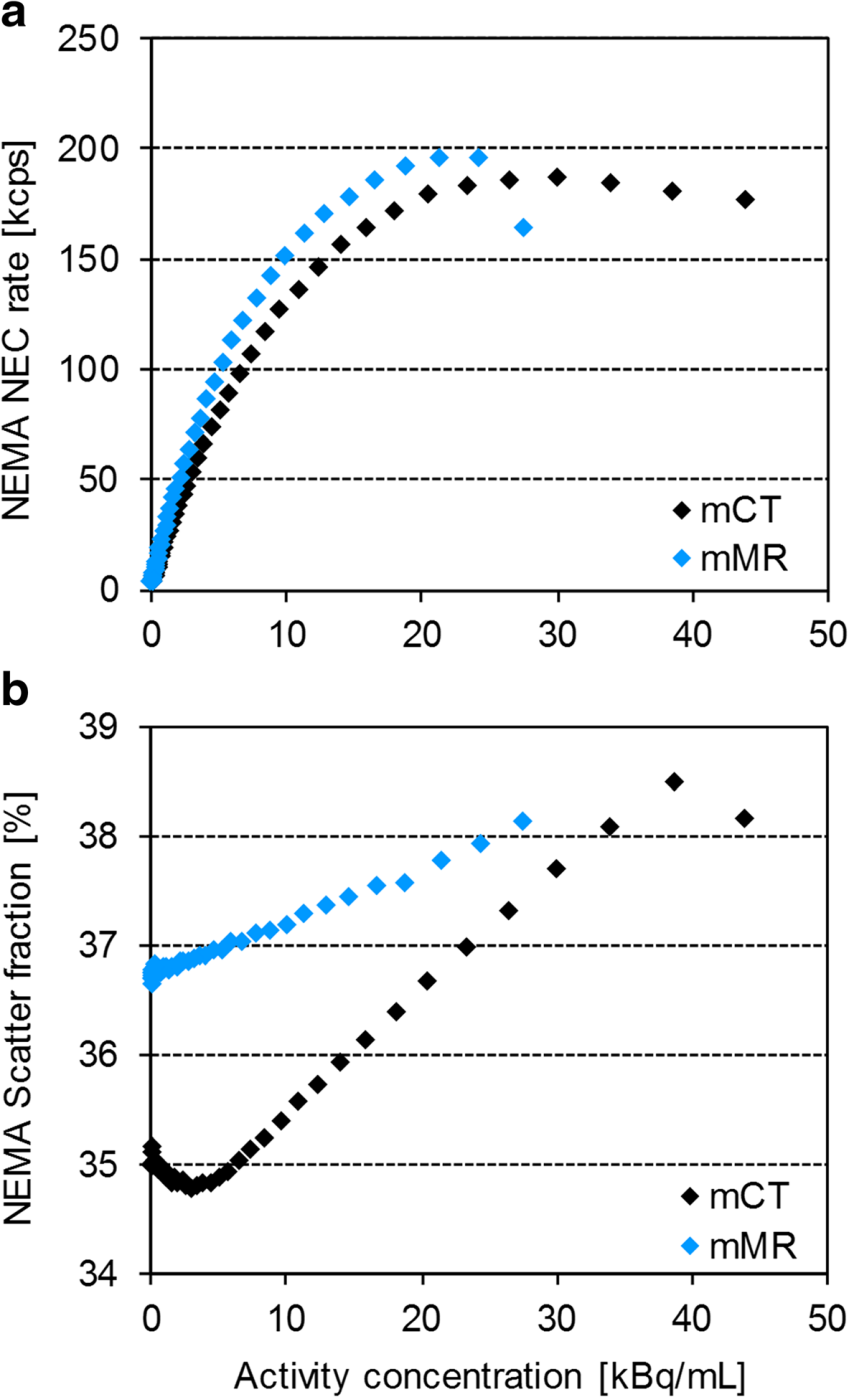
Performance measurements. NEMA NECR (**a**) and NEMA scatter fraction (**b**) for the Biograph mCT and mMR system

NECR and scatter fraction, for a range of activity concentrations, are presented in Fig. 2. For low-activity concentrations, in the clinical range, the NECR was still higher for the mMR compared to the mCT (97.9 and 78.8 kcps, respectively, at 5 kBq/mL) but also with a higher scatter fraction compared to the mCT (37.0 % vs 34.9 % at 5 kBq/mL).

Mean bias for count rate accuracy was higher for the mMR (4.9 %) than that for the mCT (1.9 %) at peak NECRs. For activity concentration in the clinical range, the mean bias was slightly higher for the mCT (1.3 %) compared to that for the mMR (0.9 %).

### Image quality

#### Contrast and background variability

Best performance (at three iterations) of the evaluated reconstruction settings resulted in approximately the same mean hot sphere contrast on both systems and a difference of 19 percentage points (pp) in mean cold contrast, in favour of the mCT for the 1:8 background-to-hot sphere activity concentration (Table 2). For the smallest sphere, the combination of PSF and TOF yielded higher contrast for the mCT (48.2 %) compared to the mMR (39.2 %). For the 1:4 background-to-hot sphere concentration, the cold sphere contrast was similar, while mean hot sphere contrast was reduced with 5 pp for both modalities (Table 3). In general, PSF increased hot sphere contrast, but not cold sphere contrast. TOF increased both hot and cold sphere contrast.

**Table 2:** Contrast results (in percent) for the NEMA phantom with various sphere sizes, with different reconstruction settings and the 1:8 ratio for the Biograph mMR and Biograph mCT

	mMR		mCT			
	OSEM	PSF	OSEM	PSF	TOF	PSF + TOF
Inner diameter (mm)						
10	36.1	39.2	35.1	41.4	36.2	48.2
13	62.1	70.2	56.7	68.8	57.1	70.3
17	73.9	79.2	64.3	72.5	67.7	74.3
22	80.5	83.0	74.2	78.3	76.7	80.7
Mean (hot spheres)	63.1	67.9	57.6	65.2	59.4	68.4
28	60.9	60.5	71.7	71.3	79.1	80.1
37	67.3	67.7	77.6	77.5	84.9	85.5
Mean (cold spheres)	64.1	64.1	74.7	74.4	82.0	82.8

**Table 3:** Contrast results (in percent) for the NEMA phantom with various sphere sizes, with different reconstruction settings and the 1:4 ratio for the Biograph mMR and Biograph mCT

	mMR		mCT			
	OSEM	PSF	OSEM	PSF	TOF	PSF + TOF
Inner diameter (mm)						
10	25.2	23.4	28.4	28.9	31.0	37.0
13	53.8	57.1	50.8	57.5	54.4	66.0
17	70.6	75.2	62.3	70.1	64.6	72.7
22	80.8	83.0	70.9	74.4	73.4	77.3
Mean (hot spheres)	57.6	59.7	53.1	57.7	55.8	63.2
28	58.7	58.8	70.8	70.9	80.3	81.0
37	67.1	67.5	76.2	76.4	84.5	85.3
Mean (cold spheres)	62.9	63.1	73.5	73.7	82.4	83.1

**Fig. 3:**
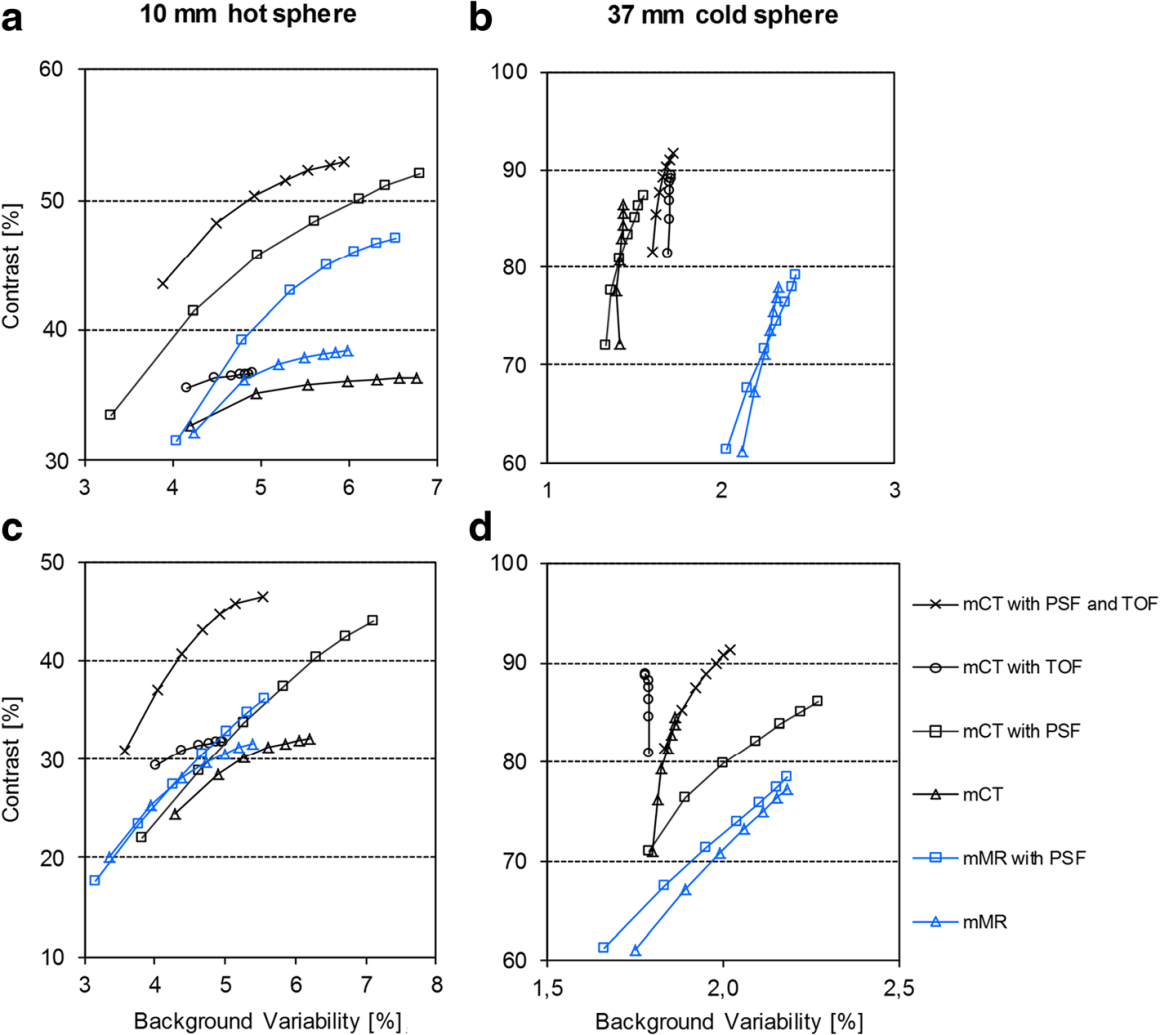
Contrast vs background variability with increasing number of iterations (2–8, *left* to *right*) and with different reconstruction settings for the Biograph mCT and mMR system; 10-mm hot sphere, 1:8 ratio (**a**), 37-mm cold sphere, 1:8 ratio (**b**), 10-mm hot sphere, 1:4 ratio (**c**) and 37-mm cold sphere, 1:4 ratio (**d**)

Contrast and background variability increased monotonically with increasing number of iterations (Fig. 3). For the lower background-to-hot sphere ratio (1:8) and the 10-mm hot sphere, the mMR and mCT yielded similar contrast values and dependency on iterations without PSF and TOF, while the results differed in favour of the mCT when taking full advantage of these additional options (Fig. 3a). A higher number of iterations for the mMR would partially compensate for the difference in contrast, but with the expense of increased background variability. For the 37-mm cold sphere, the combined effect of background variability and contrast was more clearly separated between the systems and the mCT resulted in the best performance regardless of whether PSF and/or TOF were used (Fig. 3b). For the higher background-to-hot sphere ratio (1:4), the differences between the systems became less pronounced, but still with higher contrast values in mCT images (Fig. 3c, d).

**Fig. 4:**
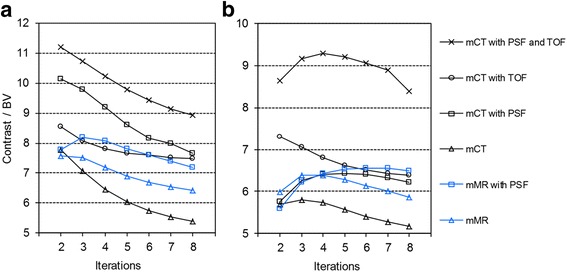
Contrast to background variability ratios for the smallest (10 mm) sphere and 2–8 iterations, with different reconstruction settings for the Biograph mCT and mMR system. 1:8 ratio (**a**) and 1:4 ratio (**b**)

Analysis of the image contrast to background variability ratio for the smallest sphere yielded best results for the mCT with PSF and TOF option (Fig. 4). For the lower background-to-hot sphere ratio (1:8), the mCT reaches best performance for two iterations and the mMR for two to three iterations, regardless of the evaluated reconstruction settings (Fig. 4a). The higher background-to-hot sphere ratio (1:4) showed a clear image improvement with the combination of TOF and PSF in the mCT reconstructed data (Fig. 4b). However, the number of iterations must be increased to reach the full potential of the systems. For reconstructions without PSF or TOF, three iterations were found optimal for both systems for the higher background-to-hot sphere ratio.

#### Lung residual error

**Fig. 5:**
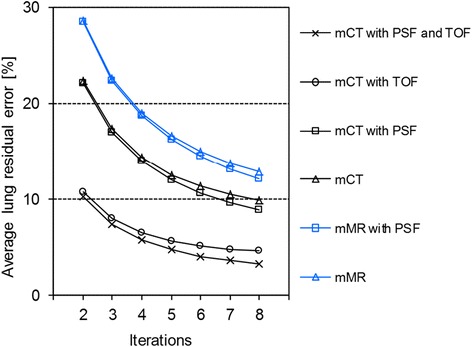
Lung residual error with different reconstruction settings for the Biograph mCT and mMR system

The lung residual error for the 1:8 background-to-hot sphere activity ratio was lower in mCT images (17 %) compared to mMR images (22 %) (three iterations, without TOF and/or PSF) (Fig. 5). No significant effect of PSF was observed. TOF resulted in a faster convergence and a lower lung residual error in general. With TOF, the lung residual error was reduced to 8 % for the mCT. The 1:4 background-to-hot sphere ratio showed similar results (data not shown).

#### SUV evaluation

**Fig. 6:**
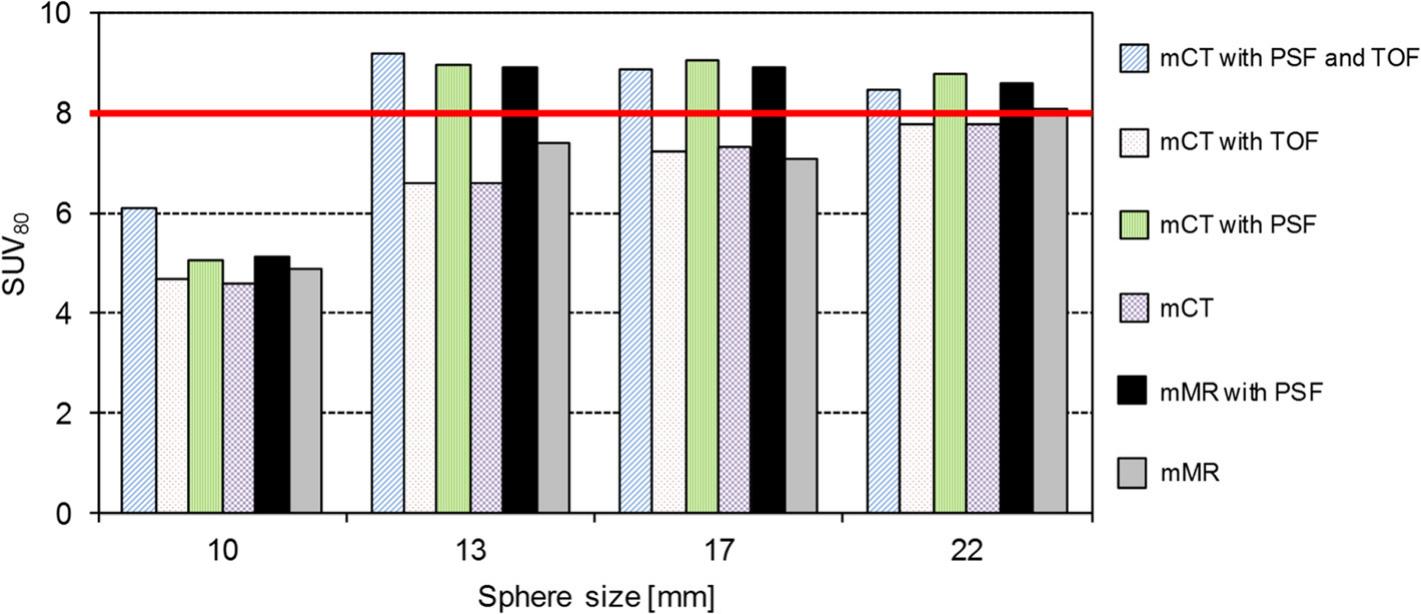
SUV_80_ for hot spheres (1:8 background to hot sphere activity concentration) and different reconstruction settings for the Biograph mCT and mMR system. The *red line* corresponds to theoretical SUV

The 22-mm hot sphere was chosen to demonstrate differences in SUV, since this sphere size could be considered to have the smallest impact of partial volume effects among the included sphere sizes. SUV_50_ underestimated the true activity concentration and SUV_max_ overestimated the values (Table 4). SUV_80_ resulted in a good estimate of the true activity concentration (within 5 % when PSF was not applied) for both background activity concentrations. There was a good correlation in SUV_80_ between the systems for all sphere sizes (Fig. 6). In general, the PSF correction overestimated the true activity concentrations. TOF did not seem to affect the quantification.

**Table 4:** SUV deviation Deviation (%) from theoretical SUV, for the 22-mm sphere, with different background concentrations and reconstruction settings for the Biograph mMR and Biograph mCT

SUV and concentrations	mMR		mCT			
	OSEM	PSF	OSEM	PSF	TOF	PSF + TOF
SUV_50_ 1:8	−15.0	−10.0	−18.8	−8.8	−18.8	−10.0
SUV_50_ 1:4	−15.0	−10.0	−22.5	−12.5	−20.0	−12.5
SUV_80_ 1:8	1.3	7.5	−2.5	10.0	−2.5	6.3
SUV_80_ 1:4	5.0	12.5	−5.0	7.5	−5.0	5.0
SUV_max_ 1:8	13.8	20.0	7.5	23.8	8.8	18.8
SUV_max_ 1:4	20.0	27.5	7.5	20.0	7.5	20.0

## Discussion

The basic performance measurements of resolution, sensitivity, count rate and scatter fraction conform well to the previously reported results by Delso et al. [18], Jakoby et al. [19] and Marti-Climent et al. [20], within experimental uncertainties. A small difference in the mMR average sensitivity can be observed in our study (13.3 kcps/MBq) compared to the study by Delso et al. (14.4 kcps/MBq). This may be explained by a significant variation in the sensitivity of the system within a few millimetres of its centre and is probably caused by a gap effect, which are unmeasured lines of response in the sinogram. In the exact centre of the FOV, this effect is less prominent than at all other projection angles. The placement of the line source in the field of view is therefore critical to minimize this effect for the mMR. For the mCT, this effect was not as prominent.

The mMR has slightly higher NECRs for both low- and high-activity concentrations compared to the mCT. The scatter fraction is approximately the same for both systems for peak NECRs. However, below peak NECR and for lower activity concentrations, which are normally used in clinical scans and for the NEMA image quality phantom, the mCT yielded lower values. This would imply that the mMR is more affected by scatter than the mCT.

Our image quality results seem to correspond well to the study by Delso et al. [18] regarding image contrast and background variability measurements for the mMR. Their results demonstrated approximately the same cold contrast (four iterations) and a somewhat lower hot contrast than ours. The lung residual error was higher in our study. Small discrepancies could be due to reconstruction and acquisition settings or minor phantom variations. The semiautomatic or manual matching of the μ-map to non-attenuation corrected PET data in the Siemens NEMA image quality software could cause some result variations because the matching depends on the researcher performing the matching/registration and not some predefined registration procedures. The new software version (Syngo MR B20P), which was used in our study, included new software features, coil modifications and other modified hardware for the Biograph mMR system, compared to the earlier systems with Syngo MR B18P version. The equivalence of these two versions has been verified and should however not affect the performance of the system [28].

For the mCT, the measured mean hot contrast was substantially higher in the study by Jakoby et al. [19] than that in our study (86.0 % vs 63.2 % when PSF and TOF were incorporated in the reconstruction), while the mean hot contrast in the study by Marti-Climent et al. [20] demonstrated approximately the same results as ours. One possible reason for these discrepancies was different reconstruction parameters. Cold contrast and lung residual errors were relatively similar between the studies.

Since the image quality results are highly dependent upon reconstruction parameters, it is difficult to compare previous studies [18–20] with each other to evaluate possible differences between the systems. For best image quality comparison between the mCT and the mMR, our study used the same reconstruction parameters, including approximately the same pixel size, the same filtration and equal number of iterations for both systems.

The mean hot contrast can be considered comparable between the mMR and mCT system. But, for the smallest sphere, the combination of TOF and PSF was in favour of the mCT. This is in accordance with the results of Akamatsu et al. [29], demonstrating that the signal-to-noise ratio for a 10-mm hot sphere was highest for the OSEM reconstruction method with a combination of TOF and PSF. This reconstruction combination also gave highest contrast recovery in the studies by Jakoby et al. [19] and Marti-Clement et al. [20] for several sphere sizes. These studies also demonstrated improved contrast recovery with TOF in cold regions, such as the lung insert and water-filled spheres, as seen in our study as well.

The average residual error, due to scatter and attenuation, was higher for the mMR than that for the mCT. This was expected due to higher scatter fraction on the mMR system. The TOF option on the mCT improved lung residual error and cold sphere contrast. In order to reduce average residual error and achieve the same cold contrast levels for the two systems, the number of iterations should be higher on the mMR than that on the mCT. Generally, the mMR performance was more dependent on the reconstruction than the mCT, and reconstruction parameters should therefore be evaluated carefully to maximize the performance of the mMR system.

SUV quantification was good for both modalities. In general, PSF correction overestimated the hot sphere values. This could be attributed to edge artefacts (Gibbs artefacts) that occur when PSF is used in iterative reconstruction algorithms, as previously shown by Nakamura et al. [30].

In this study, several problems that usually occur in the attenuation correction of clinical PET/MR data were avoided by using a CT-based template for AC of PET/MR data. Usually, uptake is underestimated in clinical PET/MR AC images due to the fact that bone is neglected in the MR AC maps [31]. Also, limitations in the MR FOV may lead to incomplete attenuation maps which cause truncation artefacts in reconstructed clinical PET images [32]. Furthermore, the flexible body surface coils are neglected in clinical PET/MR AC. Paulus et al. [33] have proven that disregarding them can lead to a bias in AC PET images that is regional dependent. The closer the analysed region is to the coil, the higher the bias. Thus, the clinical system performance will be affected by these, still unsolved, issues.

The same image quality phantom preparations were used for both system, to avoid different activity ratios between spheres and background, which may occur when preparing a phantom twice. This means that the activity concentrations were lower during the mMR acquisitions than during the mCT acquisitions. However, all image quality phantom measurements still covered normal clinical activity ranges used for these systems, as recommended in the NEMA NU 2-2007 test instructions. So this analysis should be highly relevant in comparing the clinical image quality of the two systems.

In accordance with previously reported clinical studies, demonstrating equal diagnostic performance between the mMR and mCT systems, this phantom study shows comparable performance between the scanners in most cases. However, caution should be made for more challenging settings, as for example higher background activity concentration or small uptake volumes, where the differences are more pronounced.

## Conclusions

There are detectable differences in the phantom-based performance of the Biograph mMR and mCT system. NECRs and sensitivity are higher for the mMR system. Differences in image quality are mainly related to the TOF capability of the mCT system and are most evident for the more challenging settings, where the performance is somewhat better for the mCT. However, it must be noted that the mean hot contrast for the systems are almost the same, and SUV measurements show good agreement for both systems.
